# 

**Published:** 1893-08-19

**Authors:** 


					328 THE HOSPITAL. Aug. 19, 1893.
OUB NEW OFFICES,
428, Strand, W.C., will henceforth be the Office of this Journal.
Notices of Births, Deaths, and Marriages are inserted in
this colnmn at a charge of 2s. 6d. for an announcement not exceeding
four lines.
Death.
FOSTER.?On the 11th inst., at the Evelina Hospital, of pneumonia,
Grace C. Foster (Nurse Grace), second daughter of the late Colonel
Foster Foster.
NOTICE TO CORRESPONDENTS.
All MS., Letters, Books for Review, and other matters intended for
the Editor should be addressed The Editor, The Lodge, Porchester
Square, London, W.
The Editor cannot undertake to return rejected IIS., even when
accompanied by stamped directed envelope.
Notice to Advertisers and Subscribers.
All Advertisements, Orders for Copies of The Hospital, and Business
Communications should be addressed to The Publisher, not to the Editor,
The Hospital, 428, Strand, W.C.
The Cost of Subscription, post free, is as follows Per week, 2}d.;
per quarter, 2s. 6d.; per half-year, 5s.; per annum, 8s. 6d Foreign
Per Annum, 10s. 8d.; payable, in all cases, in advance.
"Burdett's Hospital Annual," 1893.
Fourth year of publication. 700 pp. Boards, gilt lettering. A most
complete guide to British, Colonial, Indian, and American Hospitals,
Dispensaries, Nursing and Convalescent Institutions, and Asylums.
Price 5s. Published by The Scientific Press (Limited), 428, Strand,
W.C.
NOTICE.?The Index for Vol. XXXI. (ending March, 1893)
is on sale at the Office of this Journal, price 2^d.,
post free.
Scraps and Gleanings.
The Surrey Convalescent Home, neariSeaford, lias received 640 patients
in two years. The home was opened in July, 1891.
By this year's Hospital Saturday collection the receipts for the Bever-
ley Dispensary and Cottage Hospital show a falling oil of about ?30.
During the six months ending June SOth, 1893, the Free Library of St,
Martin-in-the-Fiolds was daily visited by an average of 2,368 persons.
Sports were held at the "White Horse grounds, Willesden, in aid of the
Cottage Hospital. A concert was also held later in aid of the samo
object.
The Duke of Connaught, who is general in command of the Southern
District, inspected the Medical Staff Corps at the Portsea Station Hos-
pital on Saturday. ? i
A suggestion is being taken up that the wealthy English Jews should
found a people's palace in East London which should be especially devoted
to the interests of the Hebrew community.
A demonstration of a number of friendly societies took place at South
Shields last month with the object of making known and aiding the
Convalescent Homo at Grange-upon-Sands.
The promoters of the church parade at Abingdon in aid of the funds
of the cottage hospital were somewhat disappointed at the results of their
efforts, ?10 less being collected this year than last year.
The Committee of the Hertfordshire Convalescent Home showed a
good balance-sheet for the past year's work. Lord and Lady Rothschild
have each sent ?50 towards this establishment as a donation.
It is proposed to hold a grand bazaar and ice carnival at Salisbury in
November, the proceeds of which will go towards the New Wing of tho
Infirmary. Three counties will join in the management of tho carnival.
At an examination in connection with the Sanitary Institute, held at
Cardiff last mouth, thirty candidates for the qualification of inspector of
nuisances presented themselves. Of these, sixteen obtained the qualifica-
tion.
An order has been issued at Woolwich Arsenal prohibiting operatives
from coming to their work when small-pox, scarlet fever, or other in-
fectious diseaso is present in their families or in tho houses in which they
reside.
Auxiliary courses of lectures may now be looked npon as a permanent
feature of the Edinbnrgh Dental Hospital. Six thousand two hundred
and forty-two cases of dentistry have been treated by students during tho
last six months.
A great deal of time has been spent in considering the best form of
building for the Hereford Union Infections Diseases Hospital. At the
endlof six months' discussion of various plans, it has been resolved to
erect an iron building for this purpose.
A public meeting was convened at Derby last month to consider the
necessary steps to be taken in raising ?10,000 towards the building fund
of the Royal Infirmary. Mr. Walter Evan's generous offer of ?12,000 is
dependent on the raisinglof this sum.
Mr. Ruston Peslangi Ishangir, of Her Majesty's Opium Depart-
ment, Bombay, has just published a book in defence of opium eating.
The work is chiefly composed of about 250 biographies of opium eaters,
some being soldiers, who had survived hard service.
The Eye, Ear, and Throat Hospital at Cork has found energetic
helpers in the lady visitors as well as in the Committee of Management,
who are making an earnest appeal to the public for funds to effect a
complete reconstruction of the existing building.
At the Royal Hotel, South Hackney, last week a demonstration in aid
of the Victoria Park Chest Hospital was held by the United Friendly
Trade and Temperance Societies. In five years this body of working
men has succeeded in handing over ?250 to the hospital.
Anyone wishing to give a substantial proof of their gratitude for the
able manner in which the police guarded the streets of London at the
recent Royal wedding, could most beneficially do so by sending a sub-
scription to the Convalescent Home for Police at West Brighton.
The report of the Isle of Man Hospital for the past year shows that
the receipts were fairly satisfactory and the debt rednced. The hospital
continues to receive support from all classes, and is doing a useful work.
The number of patients received during the twelve months were 333.
The annual meeting of tho subscribers of the Totnes Cottage Hospital
show' in their report receipts amounting to ?215, against an expenditure
of ?208. Mr. Arthur Champernowne has presented a piece of ground
for the erection of a new Cottage Hospital in memory of his late
father.
Attention has lately been called to the difficulty which exists for the
burning of tho bedding of infectious diseases hospitals without causing
an offensive odour to the surrounding atmosphere. Complaints of this
nature have been rife in some localities. It is to bo hoped some expedient
will soon be devised to remody this.
The Committee of Management of the Free Hospital at Kingsholm
are again indebted to the Hon. Kenneth Howard for his continued
energetic efforts in behalf of the maintenance of tho hospital, the sum of
?158 having just been collected through his exertions. Mr. Foxwell ha3
also left a legacy of ?20 to tho establishment.
Attention hni been called to the inadequate provision for the sick
and wounded on the American warships, in nearly all of which the sick-
b iy is too small, and is tucked away on the sleeping-deok, with poor light
and ventilation. A plan ifor the improved revision of these conditions
has been submitted to and approved by the authorities.
The annual demonstration of tho Deighton and surrounding districts
in aid of the Huddersfield Infirmary was held last month. The usual pro-
cession was joined by: several friendly societies, and prizes were awarded
for trade exhibits. The festivities concluded with a display of fireworks
in the evening. The total amount of money taken for tickets was con-
siderably larger than last year.
At the quarterly meeting of the Board of Governors of' the Leicester
Infirmary in July the accounts showed a deficiency of ?1,121 6s. lOd.
duo to the Treasurer, but on the whole the report bore favourable com-
parison with that of last year, the who e of ithe half-yearly income not
having yet been paid in, and nearly ?425 was to be handed over to the
funds by the Committee of the Infirmary Sports.
FIRST IMPRESSIONS OF BOOKS RECEIVED.
[It is requested that all books intended to be noticed in this column may
be sent to the Editor, at the Office, 428, Strand, W.C., not later than
Tuesday evening in each week.]
The Indian Medico Ciiiruroical Review. June 1893.
(London : Bailliere, Tindall, anil Cox.)
This review was started in January of this year, and the
number before us is therefore the sixth issue. The
pieces deresistance are an original article on Leprosy and an
abstract of the Leprosy Commission Report. The space
could not be devoted to a subject of greater interest to the
Indian reader. It is a very creditable journal.
Operation Blank. Prepared by W. W. Keen, M.D., Pro-
fessor of the Principles of Surgery in the Jefferson
Medical College, Philadelphia. Second edition.
These " Blanks " have been prepared to save time and
to ensure that all preparations are properly made for an
operation. They contain a list of directions for the nurse,
a list of dressings and medicines to be obtained. On the
level of the pad are lists of instruments required in different
operations. The idea is good, but its execution is not
specially successful.
Buxton : Its Baths and Climate : comprising a full
account of the celebrated waters and climate of Buxton,
together with special chapters on the baths, bathing,
and massage ; also excursions around Buxton and the
Peak. By Samuel Hyde, M.D., L.R.C.P., M.R.C.S.,
Physician to the Peak Hydes Thermal Establishment,
Buxton. (John Heywood, 2, Amen Corner. Second
edition.)
This is a guide to the celebrated Spa of Buxton which is
not distinguished from the commonplace guide book by
either literary or scientific merit.
Books Received.
Longmans, Green, and Co.
' A Text-Book of Domestic Economy." By F. T. Paul, F.R.C.S.
Macmillan and Co.
' Science Primers : Political Economy." By W. Stanley Jevons, F.R S.
Nichols and Co.
' Human Physiology." By T. Nichols, M.D.
John Heywood.
' Buxton : Its Baths and Climate." By Samuel Hyde, M.D.
Iliffe and Son.
' The Cyclists' Route Book." Compiled "by W. Spurrier.
Pamphlets and Periodicals.?The Westminster Review, The
Provincial Medical Journal, The Philosophy of Crime (C. H. Reeve),
The Child's Guardian, The Clinical Journal, Amateur Gardening, Bir-
mingham Medical Review, Proceedings of the Society for the Study of
Inebriety, Service of the King, Children's League of Pity, Paper, Public
Health, The Christian World, The Medical Pioneer, The Homoeopathic
World, The Health Record (London), Mission to Deep Sea Fishermen
Paper, Electro Hygiene, Invention, Westminster Hospital Medical
School.
Aug. 19,189S. THE HOSPITAL.
IX
Medical Vacancies and Appointments.
APPOINTMENTS.
P. Beach, M.B., F.R.C.P.Lond., appointed Physician for Out-
patients at the West End Hospital for Nervous Diseases,
Epilepsy, and Paralysis,Welbeck Street. H. R. Bellamy,L.R.C.S.
Edin., L.R.C.P.Edin., L.F.P.S.Glasg., appointed Honorary
Surgeon to the Scockport Infirmary. R. de la P. Beresford,
M.D.Glasg., L.R.C.P.Lond., L.R.C.P., L.R.C.S.Edin., re-
appointed Medical Officer of Health for the Oswestry Rural
ana Urban Sanitary Districts. G. F. Blacker, M.B., B.S.Lond.,
appointed Assistant Obstetric Physician at University Collpge
Hospital. W. G. H. Bradford, B.A., M.B., B.O.Cantab., ap-
pointed House Physician to the Brompton Hospital for Consump-
tion and Diseases of the Chest. W. F. Brook, F.R.C.S.Eng.,
L.S.A., appointed Medical Officer for the Throat and Ear Depart-
ment of the Swansea Hospital. A. J. Cardell,6L.D.S.R.C.S,Eng.,
appointed Honorary Dental Surgeon to the Western Dispensary,
London. J. W. Carr, M.D., B.S.Lond., M.R.C.P., F.R.C.S.,
appointed Assistant Physician to the Royal Free Hospital, Gray's
Inn Road. Dr. T. Carr, appointed Medical Officer of Health to
the Braintree Rural Sanitary Authority. A. Dixon, M.R.C.S.
Eng., L.R.C.P.Lond., appointed Medical Officer for the Eighth
District of the Preston Union. J. N. Donnellan, M.B.R.U.I.,
B.Ch., appointed Medical Officer for the Waltham St. Lawrence
District of the Cookham Union. W. T. B. Donnelly, M.B.,
B.S.Durh., M.R.C.S.Eng., L.R.C.P.Lond., appointed House
Physician to the Brompton Hospital for Consumption and
Diseases of the Chest. F. Fawssett, M.B., B.S.Lond., reappointed
Medical Officer for the outlying portion of St. John's Parish of the
Lewes Union. W. B. Fergusson, M.D., appointed Medical Officer
for the Sixth District of the Stroud Union. M. R. Gooding,
L.R.C.P.Lond., M.R.C.S.Eng., appointed Medical Officer of
Health for the Borough of Bideford. F. G. Goodman, M.D.Dub.,
L.K.Q.C.P.Irel., appointed Medical Officer for the Workhouse
and the Brigg Sanitary District of the Glandford Brigg Union.
F. Grant, L.R.C.P., L.M.Edin., M.R.C.S., reappointed Medical
Officer of Health for the iMarket Harborough Urban Sanitary
District. J. E. Halpin, L.R.C.S.Irel., L. and L.M.K.Q.C.P.Irel.,
appointed Medical Officer to the South Nor man ton Colliery Com-
C. W. Hemming, L.R.C.P., L.R.C.S.Edin.. appointed
Medical Officer for the Glyncorrwg Sanitary District of the Neath
Union. A. B. Hill, M.D.Giessen, L.R.C.P., L.M., L.R.C.S.Edin.,
D.P.H.Camb., appointed Medical Officer of Health for the ABton
Urban Sanitary District. 0. Husband, M.R.C.S., reappointed
Medical Officer of Health for the City of Ripon. R. Johnson,
M.B., B.S.Lond., F.R.C.S.Eng., appointed Assistant Surgeon to
the University College Hospital. J. Limont, M.A., M.B., B.Sc.,
M.R.C.P., appointed Physician in charge of the recently
instituted Department for Diseases of the Skin at the Royal
Infirmary, Newcastle-on-Tyne. W. G. Loveridge, L.R.C.P.,
L.R.C.S.Edin., L F.P.S.Glasg., appointed Medical Officer for
the Barton Sanitary District of the Glandford Brigg Union. W.
Loynd, L.R.C.P.I., M.R.C.S., reappointed Medical Officer of
Health for the Oswaldwistle Urban Sanitary District. H.
Lupton, L.R.C.P.Lond., M.R.C.S., reappointed Medical Officer
for the Alveston and Welford Sanitary Districts of the Stratford-
on-Avon Union. W. Mason, L.R.C.P., L.R.C.S.Edin., re-
appointed Medical Officer of Health for the St. Austall
Rural Sanitary District. G. H. Moorhead, L.R.C.P., L.M.,
L.R.C.S.Irel., appointed Medical Officer of Health for the Huns-
worth Rural Sanitary District. Mr. W. Oliver, appointed District
Medical Officer of the Township of Saddle worth. H. E.
Owen, L.R.C.P.Lond., appointed Medical Officer of
Health for the Kingsbridge District Local Board. A. M.
Palmer, L.R.C.P., L M.Edin., M.R.C.S., reappointed
Medical Officer for the Whittington Sanitary District of the
Chesterfield Union. G. R. Parker, L.R.C.P.Lond., M.R.C.S.
Eng., reappointed Medical Officer of Health for the Lancaster
Rural Sanitary District. C. E. Perry, M.D Brux., L.R.C.P.,
L.M.Edin., M.R.C.S., reappointed Medical Officer of Health for
Sandgate. T. Pimley, M.B., C.M.Aberd., appointed Medical
Officer for the Fulwood District of the Preston Union. G. W.
Pollard, M.B., C.M.Edin., appointed Junior House Surgeon to
the Borough Hospital, Birkenhead. W. Ridley, M.B., M.S.,
F.R.C.S., appointed Surgeon in charge of the recently instituted
Department for Diseases of the Throat and Ear at the Royal
Infirmary, Newcastle-on-Tyne. Q. Renton, M.D.Edin., ap-
THE HOSPITAL. Aug. 19, 1893.
pointed Medical Officer of Health for the Leadgate Urban
Sanitary District. T. W. Shortridge, M.D.Brux., L.R.C.P.,
L.R.C.S.Edin., reappointed Medical Officer of Health for Honi-
ton. H. E. Spencer, M.D.Lond., appointed Professor of Mid-
wifery and Obstetric Medicine at University College, and
Obstetric Physician at University College Hospital. J. W.
Stephens, L.R.C.P.Lond., M.R.C.S.Eng., appointed Certifying
Snrgeon under the Factory Acts for the Cardigan District. F.
Stephenson, L.F.P.S.Glasg., L.R.C.P.Edin., reappointed Medical
Officer of Health for the Prestwich Urban Sanitary District. F.
Stockwell, M.D.Lond., M.R.C.S., reappointed Medical Officer of
Health for tlie Wincanton Rural Sanitary District. A. W.
Thomas, L.R.C.P., L.R.C.S.Edin., appointed Medical Officer
for the Soorle Sanitary District and the Workhouse of the
Swaffham Union. F. C. Warren, L.R.C.S., L.M.Edin., reap-
pointed Medical Officer of Health for Gillingham. R. T. Williams.
L.R.C.P., L.R.C.S.Edin., L.F.P.S.Glasg., appointed Medical
Officer of Health for the Porthcawl Local Board. C. Wilson,
M.B., C.M.Glasg., appointed House Surgeon to the Wirral
Children's Hospital, Birkenhead. Mr. J. Young, appointed
Medical Officer for the Fourth District of the St. Alban's
Union.
VACANCIES.
The following vacancies are announced this week, the amount of
salary in each case being given after the name of the institution :
Befcident Medical Officers.
Birmingham City Asylum, Birmingham. Junior Assistant Medical
Officer. Salary ?80 per annum, with board, lodging, and washing.
Applications to the Medicnl Superintendent.
Brighton, Hove, and Preston Dispensary, Board Room, Queen's Road,
Brighton. House Surgeon, unmarried, doubly qualified. Salary
?140 per annum, with furnished apartments, coal, gas, and
attendance. Applications to 0. Somers Clarke, Honorary Secretary,
by September 1st.
Cumberland Infirmary, Carlisle. Assistant House Surgeon. Salary ?40
per annum, with board, lodging, and washing. Applications and
testimonials to the Secretary by August 17th.
County Asylum, Lancaster. Clinical Assistant. Board, &c., provided.
Applications to the Medical Superintendent.
General Hospital, Birmingham. House Physician. Salary ?70 per
annum, with residence, board, and washing. Applications to Howard
J. Collins, House Governor, by September 2nd.
London County Asylum, Claybury, "Woodford, Essex. Fourth Assistant
Medioal Officer, unmarried, doubly qualified, and not more than 35
years of age. Salary ?150, rising ?5 a year to ?160 per annum.
Applications on specified form to R. W. Partridge, Clerk to the
Asylums Committee, 21, Whitehall Place, S.W., "by August 24th.
London Temperance Hospital, Hampstead Road, N.W. House Surgeon
and Junior House Surgeon, doubly qualified. Residence, board, and
washing provided. Appointments for six months. The House
Surgeon will receive a salary at the rate of 50 guineas per annum,
and the Junior House Surgeon will receive an honorarium of 5
guineas on satisfactory completion of term of appointment. Appli-
cations to E. Wilson Taylor, Secretary, by August 31st.
Radcliffe Infirmary, Oxford. House Physician, doubly qualified. Salary
?80 per annum, with board, lodging, and washing. Appointment
for two years. Applications to the Secretary by August 18th.
Royal United Hospital, Bath. House Surgeon; must be M.R.O.S.Eng.
Salary ?60 per annum, with board, lodging, and washing. Applica-
tions^ W. Stockwell, Secretary-Superintendent, by August 25th.
St. Luke's Hospital, London, E.G. I Clinical Assistant. Appointment
for six months. Board and residence provided. Applications to
Percy de Bathe, Secretary, by August 24th.
Teignmouth, Dawlish, and Newton Infirmary, Dispensary, and Con-
valescent Home. House I Surgeon, doubly qualified. Salary ?60
per annum, with board, lodging, and washing. Applications to the
Chairman of Committee by August 28th.
Wolverhampton and Staffordshire General Hospital, Wolverhampton.
House Surgeon. Salary ?100 per annum, with board, lodging, and
washing. Appointment for one year, but eligible for re-election.
Applications endorsed " Application for House Surgeon " to Edwin
A. White, House Governor and Secretary, by August 28th.
Worcester County and City Lunatic Asylum. Third Assistant Medical
Officer, unmarried. Salary ?100 per annum, with board, lodging,
and washing. Applications to Dr. Cooke, The Asylnm, Powick, near
Worcester, by August 25th.
Non-Besicleiit Medical Officers.
Anderson's College Medical School, Glasgow. Chair of Materia Medica
and the Chair of the Physics. Applications to John Kidstone, Secre-
tary, 50, West Regent Street, Glasgow, by August Slst.
National Dental Hospital, Great Portland Street, W. Assistant
Dental Surgeon. Applications to E. Almack, Secretary, by August
22nd.
Queen's College, Ireland. Professor of Midwifery. Applications to the
Under-Secretary, Dublin Castle, by August 21st.
Westminster Hospital, Broad Sanctuary, S.W. Surgeon. Must be
Fellow of the Royal College of Surgeons of England. Applica-
tions, with testimonials, to the Secretary, S. M Quennell, by August
22nd.

				

